# Diarrheal diseases burden and causes in an Infectious Diseases department during the era of *Clostridium difficile* infection

**DOI:** 10.1186/1471-2334-14-S7-P82

**Published:** 2014-10-15

**Authors:** Gabriel Adrian Popescu, Diana Tănase, Dana Bunescu, Adrian Abagiu, Georgeta Preda, Alice Atomoaie, Florin Dună

**Affiliations:** 1 Carol Davila University of Medicine and Pharmacy, Bucharest, Romania; 2 National Institute for Infectious Diseases "Prof. Dr. Matei Balş", Bucharest, Romania

## Background

Classically, the diarrheal diseases were a top diagnosis in Romanian infectious diseases hospitals during summer. The increasing incidence of *Clostridium difficile* infection (CDI) after 2011 might change this seasonality of diarrheal diseases, as warm season illnesses. Aim: to estimate the burden of different diarrheal diseases in an Infectious Diseases department and to evaluate if the seasonality still exists.

## Methods

We analyzed the monthly distribution of admissions in the Sixth department of the National Institute for Infectious Diseases "Prof. Dr. Matei Balş" during February-July 2014; the burden of diarrheal diseases was estimated as percentage of total patient x hospitalization days. Diarrheal diseases were divided in five subgroups: CDI, food poisoning with quick recovery, infectious diarrhea with identified etiology, diarrhea as symptom in other diseases, unknown etiology diarrhea.

## Results

A number of 110 patients with diarrhea were hospitalized in the analyzed period, cumulating 852 patients x hospitalization days, 25.2% of total hospitalization days. No seasonality for burden of diarrheal diseases was discerned; higher burden were reported for months of March 31.7%, RR=1.32 (1.14; 1.53), p=0.0003 and July, 38.9% RR=1.72 (1.51; 1.95), p<0.0001, and lower level in February 12.2%, CI95% (9.4%; 15.6%).

The patients from the five subgroups represented: CDI, 52 of patients (47.3%) and 66% of patients x days; food poisoning, 13 patients and 2.9% of hospitalization days; other infectious diarrhea (*Salmonella*, rotavirus), 4 patients and 2.8% of hospitalization days; unknown etiology diarrhea, 31 patients and 22.2% of hospitalization days. For 10 patients summing 52 hospitalization days (6.1%), diarrhea was an indicative symptom for different diseases, including serious ones: colorectal neoplasia – 3 patients, Crohn’s colonic disease – one patient, periappendicular abscess – one patient, severe methotrexate mucositis – one patient, colchicine side effects – one patient.

## Conclusion

CDI has the highest burden among diarrheal diseases in the Infectious Diseases Department; the CDI emergence seems to eliminate the seasonality of diarrheal diseases. A small number of admissions are avoidable with a short-stay system. In diarrheal diseases with prolonged evolution and unknown etiology several serious causes need to be eliminated: colorectal neoplasia and inflammatory bowel diseases, severe post-drug side effects, surgical diseases.

